# Cutting Through History: The Evolution of Glioblastoma Surgery

**DOI:** 10.3390/curroncol31110485

**Published:** 2024-10-24

**Authors:** Abdullah H. Ishaque, Sunit Das

**Affiliations:** 1 Division of Neurosurgery, Department of Surgery, University of Toronto, Toronto, ON M5S 1A1, Canada; abdullah.ishaque@mail.utoronto.ca; 2 Division of Neurosurgery, Department of Surgery, St. Michael’s Hospital, Toronto, ON M5B 1W8, Canada

**Keywords:** glioblastoma, surgery, history

## Abstract

Despite significant advancements in neuro-oncology, management of glioblastoma remains a formidable challenge. Over the last century, the role and goals of surgery for patients with glioblastoma have evolved dramatically, with surgical intervention maintaining a central role in patient care. To understand the future role of surgery in the management of glioblastoma, we must review and appreciate the historical journey that has led us to this juncture. Here, we provide an overview of this evolution and speak about anticipated changes in the future. “*Certainly we cannot hope to solve the glioblastoma problem by throwing up our hands and saying there is nothing we can do. On the contrary, the solution lies in our constantly pressing on, making more and more strenuous efforts to remove these tumors, and not allowing ourselves to be deterred by any obstacles that lie in our path.*”—Ernest Sachs, 1950.

## 1. Introduction

Glioblastoma is the most aggressive and lethal primary brain tumor in adults, known for its rapid progression and resistance to treatment. Despite significant advancements in neuro-oncology, glioblastoma remains a formidable challenge. Over the last century, the management of these tumors has evolved dramatically, with surgical intervention maintaining a central role in patient care. Early surgical approaches primarily aimed to decompress the brain to alleviate the pressure caused by the tumor. As our understanding of neuroanatomy and tumor biology has progressed, surgical strategies have shifted toward more targeted operations focused on precise tumor resection.

The advent of technological innovations, such as advanced imaging methods like computed tomography (CT) and magnetic resonance imaging (MRI), have transformed the field by enabling more accurate tumor localization. This has dramatically improved both surgical safety and patient outcomes. Molecular and genetic characterization of tumors has further revolutionized treatment by allowing individualized and tailored therapeutic approaches that optimize patient care.

Today, treatment for glioblastoma involves a multidisciplinary team, including neurosurgeons, neuro-oncologists, radiation oncologists and neuropathologists. Surgical goals have expanded from simple decompression to obtaining tissue for diagnosis and performing maximal safe resection aimed at reducing tumor burden while preserving critical neurological function. The integration of advanced surgical tools, such as 5-aminolevulinic acid (5-ALA) fluorescence-guided resection and intraoperative neuro-navigation, has enhanced the surgeon’s ability to achieve complete resections and, consequently, improve patient survival and quality of life.

This paper explores the rich history of glioblastoma surgery, tracing its development from the earliest days of neurosurgery to the modern era of precision techniques and multimodal treatment. Understanding the historical context of glioblastoma management is essential for advancing patient care, helping clinicians and researchers appreciate how far the field has come. This paper also considers future directions in neurosurgery, with a particular focus on personalized medicine and technological advancements.

## 2. Establishing the Role of Surgery in Glioma Treatment

Rudolf Virchow is credited as the first to pathologically describe the term “glioma”. In the 1850s, he described the term and concept of neuroglia—connective tissue or “nerve-glue”—in a series of lectures in Berlin [1]. He classified gliomas as tumors that arose from the connective tissue of the central nervous system. Case studies of gliomas from the late 19th century were primarily autopsy reports detailing patient presentations and clinical courses, as treatment was primarily supportive. Management of these patients was typically limited to clinical encounters during significant events such as seizures, focal weakness or changes in levels of consciousness [2,3]. Physicians at the time grappled with the fundamental question of neuroanatomical localization of pathology in their patients, which perplexed them not only during clinical evaluations, but also during surgery, when attempted.

**Figure 1:**
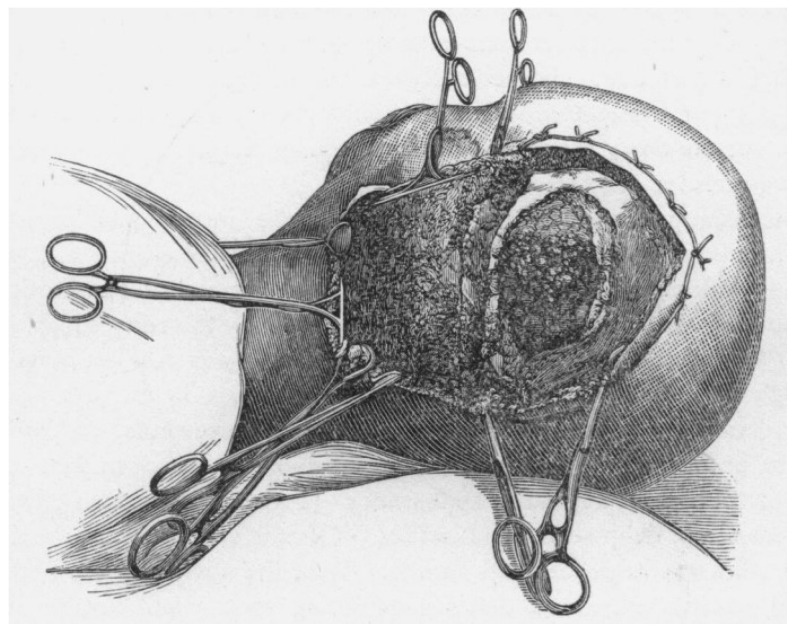
Illustration depicting the skin incision and craniotomy performed in the late 1890s for surgery for a cerebral tumor. Illustration taken from Pilcher (1898). Reprinted with permission from Ref. [4]. Copyright (2024), Wolters Kluwer Health, Inc.

**Figure 2:**
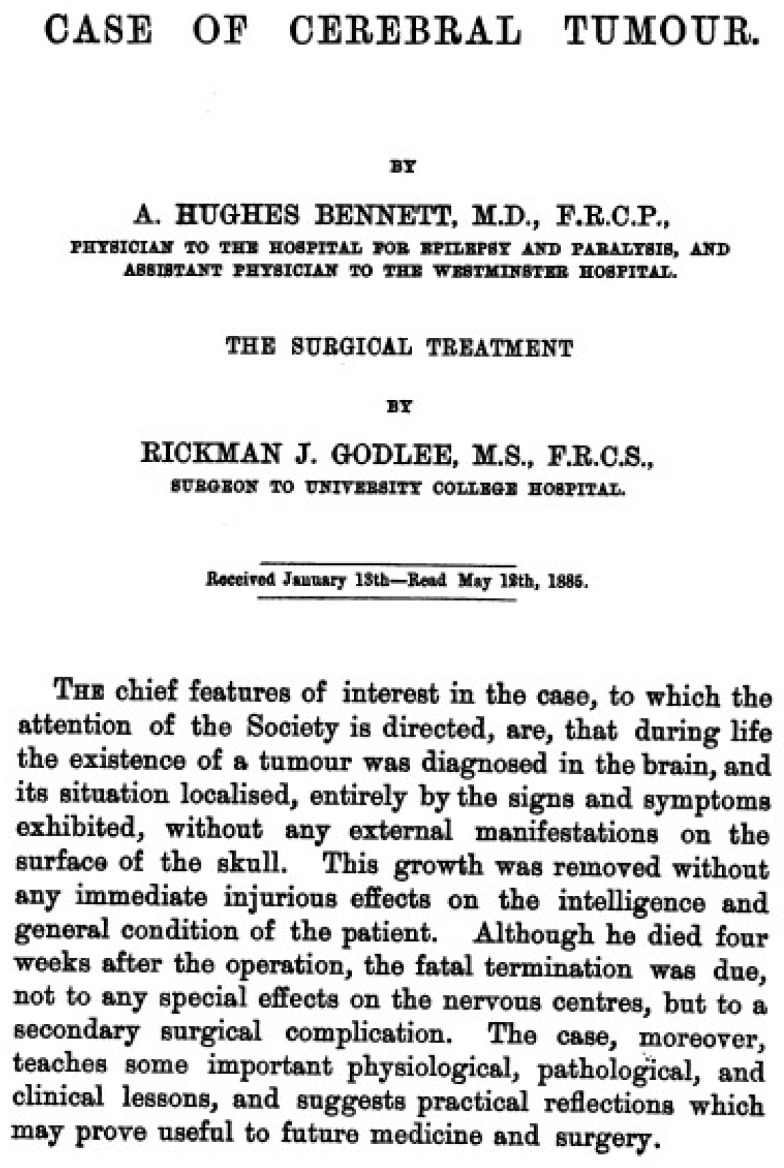
The first manuscript detailing the resection of a glioma in a 25-year-old patient was published in 1884 by Bennett and Godlee. Adapted from Ref. [5]. No copyright permission required as publication is older than 70 years.

Surgeries for gliomas were exploratory procedures involving large craniotomies. Lesions were localized by passing needles into the brain in various directions to assess texture and pressure (Figure 1) [4]. The first reported case of tumor resection was performed by Rickman Godlee in 1884 (Figure 2). The patient was a 25-year-old male who suffered from progressive symptoms of seizures involving the left side, weakness, headaches and intractable vomiting. The medical team concluded that the patient likely had a small brain tumor involving the cortex in the upper third of the Rolandic fissure. During the operation, no cortical lesion was discovered; however, the surgeons followed the blood vessels and discovered a “hard glioma” about a quarter of an inch deep to the surface, which they resected. The case highlighted the challenges faced by surgeons in the late 19th century [5]. Not only were surgical techniques limited and the localization of lesions challenging, but postoperative care was plagued by high mortality rates driven primarily by infection. The patient recovered initially, but soon succumbed to meningitis and cerebral herniation.

Despite early attempts, many remained skeptical about the role of surgery in patients with brain tumors. Horatio Wood, a Pennsylvanian surgeon during the American Civil War, believed that surgical resection was “practicable in about three percent of the cases” and “justifiable only when the tumor is clearly located in the psycho-motor zone” [6]. His infamous remark that brain surgery “has been very valuable to neurology by affording early post-mortem” reflects the pessimism surrounding surgical intervention at the time. Reaching a diagnosis of brain tumor was often delayed—frequently made only when symptoms of increased intracranial pressure (headaches, vomiting and visual disturbances) became severe—and combined with inadequate treatment; it was an intervention that nearly uniformly led to a poor outcome. Philip Knapp, a Boston neurologist, noted in 1899 that in 20% of operations, failure to remove the tumor was a direct result of error in diagnosis [7]. He additionally acknowledged that gliomas are infiltrating lesions and that about a third of patients die from tumor recurrence [7].

Victor Horsley emerged as one of the key proponents of early surgical intervention for brain tumors. He argued that the progressive nature of symptoms—such as motor deficits and seizures—was a critical indication for surgery, even before more severe signs of raised intracranial pressure appeared [8]. Horsley believed that the prevailing practice of delaying surgery in favor of medical management was detrimental to patients, and suggested that medical treatment with drugs such as potassium iodide should not extend beyond six weeks if there was no significant improvement. However, Horsley acknowledged that surgery for gliomas was largely palliative, though he attributed this to delays in diagnosis and intervention. He was also one of the early surgeons to document surgical techniques; he proposed staged procedures to reduce the incidence of shock and packing the surgical cavity to reduce postoperative hemorrhage [8]. Enthusiasm about neurosurgical interventions in brain tumors was slowly on the rise as the collective surgical experience reported a decline in postoperative mortality over time [9].

## 3. Evolving Surgical Goals: From Palliation to Improving Survival

One of the primary goals of neurosurgical intervention in the late 19th and early 20th centuries was surgical decompression to manage raised intracranial pressure caused by tumor growth. Horsley, in his discussion of the treatment of brain tumors, advocated for “opening of the skull… for the purpose of relieving these symptoms” [8]. He believed that if medical management failed, “opening of the skull”—or craniectomy—should be performed even in cases when the tumor could not be removed. This concept was further solidified and explained by Harvey Cushing. In his 1920 address on updates in the then budding field of neurosurgery, he noted that “unexpected amelioration of subjective symptoms had taken place” even in cases where no tumor was found on surgical exploration [10]. Papilledema was therefore not due to optic neuritis, which was at the time labeled as one of the cardinal signs of brain tumors and increased intracranial pressure, but rather from raised intracranial pressure itself. He further proclaimed that subtemporal and suboccipital decompressions are two of the most important neurosurgical procedures, even in cases in which tumors were not found.

To shift the surgical goal from merely decreasing intracranial pressure to providing a survival benefit to the patient, a deeper knowledge of histopathology and tumor classification was paramount. In 1912, Howard Tooth published the first detailed clinico-histopathological study of 127 confirmed gliomas. In his series, he characterized gliomas based on their location and found the average survival length of patients with forebrain gliomas to be 10 months, and 9 months for patients with tumors in the brainstem [11]. He also attempted to characterize the stages of glioma growth, which he hypothesized began with a pre-malignant stage. Tooth proposed that active proliferation was explained by an interplay between the nucleus, glial cells and blood vessels. Following this work, Joseph Globus and Israel Strauss further characterized glioblastomas as being highly cellular with necrotic areas that cause rapid functional decline and short survival [12]. Perhaps most famously, Percival Bailey and Cushing characterized glial tumors—and used the term glioblastoma multiforme—based on their histology and correlated them to patients’ survival in a landmark publication in 1926 [13].

Reports began to emerge of proposed strategies for the surgical management of glioblastomas. Kenneth MacKenzie, a prominent neurosurgeon in Toronto, believed that the optimal surgical strategy was to resect the tumor sufficiently to leave a large surgical cavity [14]. He coined this approach “internal decompression” and believed that it would allow patients to live for several months with sustained quality of life. Interestingly, he noted that when managing astrocytomas, which Bailey and Cushing had earlier classified, only a subtemporal decompression was necessary, as astrocytomas had a better prognosis compared to glioblastomas [14]. For glioblastomas, he believed that subtemporal decompression was a morbid procedure that did not improve quality of life. In essence, his dogma was to preserve quality of life and to perform surgical resection of the tumor if prolonging meaningful life was attainable. Extending from the idea of internal decompression, Walter Dandy, perhaps best known for his introduction of ventriculography as a means to localize intracranial lesions, published a case series of five patients describing his experience of performing right-sided hemispherectomy in patients with gliomas [15]. Dandy was an advocate for early and aggressive surgery for gliomas, though there are no records of his outcomes.

Davis et al., in their 1949 publication, provided the first comprehensive clinical series documenting histology, surgical technique and survival outcomes [16]. They argued for large craniotomies to allow for exposure, total tumor resection and decompression of the brain. They showed that 50% of patients with glioblastoma who underwent a lobectomy survived for longer than one year. Comparatively, only 26% of patients who underwent complete resection of visible tumor survived for more than a year, and only 18–21% of the patients who underwent biopsy or subtotal resection survived for more than a year [16]. Over the next decade, several other studies were published that investigated large case series of glioblastoma patients and the impact of the extent of resection on survival. In a study of 506 glioblastomas from the Montreal Neurologic Institute, the authors concluded that patients who underwent complete removal survived longer than those who underwent incomplete removal [17]. Similarly, a group from Yale reported longer survival rates in glioblastoma patients undergoing total resection versus partial resection or biopsy alone [18]. At the same time, there was rising evidence that radiation therapy also provided a survival benefit to patients with glioblastoma [17,18,19]. Surgical outcomes were greatly improved and intraoperative mortality rates reduced by technological innovations such as electrocautery, cerebral angiograms and ventriculography. By the 1970s, there was mounting evidence to advocate for a multimodal approach to treating glioblastoma: tumor resection with the goal of total resection followed by radiotherapy provided better outcomes.

The advent and widespread use of MRI propelled research and clinical practice, as preoperative tumor localization and postoperative measurements of the extent of resection became standard practice. In a landmark MD Anderson-led study that investigated the prognostic value of the extent of resection using MRI in a series of 416 patients with glioblastoma, there was a clear survival benefit of more than four months in patients with greater than 98% tumor resection [20]. The study acknowledged that the extent of tumor resection is dependent on the initial tumor size and location. Tumors in eloquent areas posed a significant challenge. Mitch Berger from the University of California, San Francisco, added to this growing knowledge base by demonstrating a near-linear relationship between extent of resection and increased survival in 500 GBM patients [21]. Tumors adjacent to or within eloquent areas, particularly language and motor areas, pose significant challenges to attaining gross total resection while maintaining neurological function. Berger and his group extensively studied gliomas in eloquent areas and established the safety and efficacy of awake craniotomies to monitor function and prevent postoperative neurological deficits [22,23]. The preservation of neurological status is of critical importance, as there is evidence that patients with glioblastoma who suffer new neurological deficits after resection have worse survival outcomes [24]. These findings put a spotlight on the current neurosurgical goals of achieving safe and maximal resection for a survival benefit in patients with glioblastoma.

## 4. Establishing Multimodal Therapy and Maximal Safe Resection

The years that followed saw a substantial amount of effort to investigate various iterations and combinations of chemotherapy and radiation therapy for the treatment of patients with glioblastoma. Notable trials were reported from the Brain Tumour Cooperative Group [25], the Radiation Therapy Oncology Group, the Eastern Cooperative Oncology Group [26] and the Northern California Oncology Group [27]. A key breakthrough in the utility of chemotherapy for glioblastoma treatment was the identification of alkylating agents, in particular temozolomide. Most notably, Stupp et al. performed a phase II study in 2002 investigating the use of temozolomide concomitantly with postoperative radiotherapy that demonstrated its safety and efficacy [28]. This was followed by the landmark study in 2005 that established the current standard of care for patients diagnosed with glioblastoma. Stupp et al. demonstrated a two-year survival rate of nearly 27.2% compared to 10.9% with the previous standard of care of surgery and post-operative radiotherapy alone [29]. As critically, the authors found that the addition of temozolomide chemotherapy to surgery and radiation improved 5-year survival from 1.9% to 9.8%, compared to patients treated with surgery and radiation therapy alone [30]. In their publication, they noted that standard therapy at the time was “surgical resection to the extent that is safely feasible”. This highlighted the prevailing cautious approach of the time with respect to surgical technique and the degree of resection that should be targeted.

## 5. The Present: Going Beyond Just the Tumor

The vast body of neurosurgical literature now supports the current standard of care of maximal safe surgical resection for patients with glioblastoma. Surgical techniques have similarly advanced to achieve this goal even in the most precarious locations in the brain. Insular gliomas pose a formidable challenge, as they are surrounded by critical structures including language and motor centers, lenticulostriate arteries and the Sylvian vessels. The Sanai–Berger Insular Glioma Classification system was developed and validated by the group at the University of California, San Francisco, to anatomically classify insular gliomas and guide surgical resection strategies [31,32]. Tumors in the anterior superior region, or Zone 1, had the largest extents of resection, whereas tumors in the posterior regions were limited by the motor structures. Evidence again demonstrated that the extent of resection, even in the insular region, is predictive of overall survival in patients suffering from glioblastoma [31,32].

Technological advancements and our understanding of anatomy and neurophysiology have also substantially increased over recent times, allowing neurosurgeons to push the boundaries of “safe” resection. Advances in MRI have enabled us to capture the anatomical basis of various cognitive functions, such as language and motor tasks, to tailor surgical resections for each patient [33,34]. Similarly, diffusion tensor imaging has proved to be an instrumental technique in preoperative planning for tumor resection. With this technology, surgeons can map the white matter pathways in the vicinity of the tumor or any lesion prior to a surgical approach to preserve the integrity of important axons [35]. Notably, tumor surgery, particularly glioma surgery, has contributed significantly to the neuroscience literature. Combining data from intraoperative neuropsychological assessments during awake craniotomies and preoperative neuroimaging studies led to insights into brain plasticity [36,37,38], and language function and anatomical correlates have been discovered and often revised older beliefs [37,39]. In addition to neuroimaging techniques and intraoperative neuropsychological assessment, intraoperative neuronavigation and tumor visualization adjuncts have also revolutionized neurosurgical treatments for gliomas. Intraoperative neuronavigation in the current era has certainly eradicated the problem of localizing the lesion. Moreover, studies have demonstrated a survival benefit with the use of intraoperative neuronavigation systems to guide complete resections [40]. Other adjuncts that have been used to improve intraoperative anatomical guidance for glioma resection include intraoperative ultrasound and intraoperative MRI [41]. The use of 5-ALA, a non-fluorescent prodrug that leads to intracellular fluorescence in GBMs that is visualized intraoperatively, has also significantly increased in the last two decades. A phase III clinical trial demonstrated more complete resections with the use of 5-ALA in microscopic surgical resections of GBMs, leading to a significant survival benefit [42]. A follow-up phase II study in recurrent malignant gliomas also demonstrated its efficacy despite the use of prior radiation or chemotherapy [43]. This progress led the field to consider the prospect of supramaximal resection, with initial efforts suggesting a further survival benefit with this approach [44,45,46].

The stagnancy in improving the survival of patients with glioblastomas has motivated and led to the implementation of unique technological advances. MRI-guided laser-induced interstitial thermotherapy (LITT) has emerged as a tool in the management of patients suffering from recurrences of GBM or deep-seated tumors [47]. The central principle of LITT revolves around the increased susceptibility of tumor cells to thermal injuries. The safety of this relatively novel technology has been established in numerous studies [48,49]. Although initial experience demonstrating a substantial survival benefit of using LITT in glioblastoma is lacking, studies have recently discovered that this technology enables opening of the blood–brain barrier (BBB) [50,51]. This is of critical importance because the BBB has largely been an obstacle for effective delivery of chemotherapeutic agents in neuro-oncologic cases. Similarly, another novel technology has had early success with opening the BBB: studies using focused ultrasound (FUS) have shown clear evidence that FUS can disrupt the BBB and increase its permeability [52,53]. Neurosurgical applications of FUS are well established in functional neurosurgery for the treatment of essential tremors and Parkinson’s disease. With mounting evidence of the utility of FUS and LITT in neuro-oncology, these technologies have opened the doors for opportunities to improve the delivery of chemotherapeutic agents in the treatment of glioblastomas.

## 6. The Future of Glioblastoma Surgery

The history of glioblastoma surgery has evolved dramatically since its beginnings in the late 19th century. From craniectomies to gross total resection, neurosurgeons have navigated through phases of pessimism and optimism. Certainly, the role of the neurosurgeon and microsurgical resection of gliomas has been securely established over time, as mounting evidence supports the need for radical tumor resection. No doubt, neurosurgical knowledge works in tandem with genetic and molecular discoveries as we continue to learn the various biological predictors of outcomes in glioblastoma [54,55]. Advances in our understanding of the underlying biology are dependent on the availability of human tumor tissue, and this has cemented neurosurgeons’ role not only in the clinical realm, but also in the research realm of glioblastomas [56,57,58]. Availability and research on tissue samples will accelerate the discovery of targeted, molecular therapies of this devastating disease. As Ernest Sachs observed in 1950, “the sound method of dealing with the glioblastomas seems… to continue to attempt to remove these tumors surgically, always aiming to be radical enough to try to obtain a cure”. This sentiment still holds true today. Until more effective therapies are developed, neurosurgeons will continue to play a crucial role in the management of GBM, striving to alleviate symptoms and extend survival where possible. The challenges ahead are significant, and a multidisciplinary effort involving surgical innovation, molecular biology and novel therapeutics will be essential to make meaningful progress against this formidable disease.
